# Variation in Urinary Flow Rates According to Demographic Characteristics and Body Mass Index in NHANES: Potential Confounding of Associations between Health Outcomes and Urinary Biomarker Concentrations

**DOI:** 10.1289/ehp.1408944

**Published:** 2015-01-27

**Authors:** Sean M. Hays, Lesa L. Aylward, Benjamin C. Blount

**Affiliations:** 1 Summit Toxicology, LLP, Lyons, Colorado, USA; 2 Summit Toxicology, LLP, Falls Church, Virginia, USA; 3 National Research Centre for Environmental Toxicology (Entox), University of Queensland, Brisbane, Queensland, Australia; 4 National Center for Environmental Health, Centers for Disease Control and Prevention, Atlanta, Georgia, USA

## Abstract

**Background:**

Urinary analyte concentrations are affected both by exposure level and by urinary flow rate (UFR). Systematic variations in UFR with demographic characteristics or body mass index (BMI) could confound assessment of associations between health outcomes and biomarker concentrations.

**Objectives:**

We assessed patterns of UFR (milliliters per hour) and body weight–adjusted UFR (UFRBW; milliliters per kilogram per hour) across age, sex, race/ethnicity, and BMI category in the NHANES (National Health and Nutrition Examination Survey) 2009–2012 data sets.

**Methods:**

Geometric mean (GM) UFR and UFRBW were compared across age-stratified (6–11, 12–19, 20–39, 40–59, and ≥ 60 years) subgroups (sex, race/ethnicity, and BMI category). Patterns of analyte urinary concentration or mass excretion rates (nanograms per hour and nanograms per kilogram per hour BW) were assessed in sample age groups for case study chemicals bisphenol A and 2,5-dichlorophenol.

**Results:**

UFR increased from ages 6 to 60 years and then declined with increasing age. UFRBW varied inversely with age. UFR, but not UFRBW, differed significantly by sex (males > females after age 12 years). Differences in both metrics were observed among categories of race/ethnicity. UFRBW, but not UFR, varied inversely with BMI category and waist circumference in all age groups. Urinary osmolality increased with increasing BMI. Case studies demonstrated different exposure–outcome relationships depending on exposure metric. Conventional hydration status adjustments did not fully address the effect of flow rate variations.

**Conclusions:**

UFR and UFRBW exhibit systematic variations with age, sex, race/ethnicity, and BMI category. These variations can confound assessments of potential exposure–health outcome associations based on urinary concentration. Analyte excretion rates are valuable exposure metrics in such assessments.

**Citation:**

Hays SM, Aylward LL, Blount BC. 2015. Variation in urinary flow rates according to demographic characteristics and body mass index in NHANES: potential confounding of associations between health outcomes and urinary biomarker concentrations. Environ Health Perspect 123:293–300; http://dx.doi.org/10.1289/ehp.1408944

## Introduction

Biomonitoring has been called “the gold standard” for chemical exposure assessment in environmental epidemiology (Sexton et al. 2004), and use of biomonitoring in such studies has expanded rapidly. The direct measurement of the chemical of interest in biological samples from individuals within a study population provides powerful information in the study of chemical exposures. However, valid interpretation of the biomonitoring data, particularly in the study of potential associations with health outcomes, will be enhanced by increasing understanding of the various physiological characteristics, temporal and pharmacokinetic issues, and other factors that might potentially affect the relationship between measured biomarker concentration and relevant chemical exposure levels.

The U.S. National Health and Nutrition Examination Survey (NHANES) cross-sectional data sets have proven to be a rich database allowing development of hypotheses regarding exposures to environmental chemicals (as reflected in biomonitoring data) and health outcomes. One area of particular interest has been the evaluation of chemicals with potential endocrine activity. Several studies have examined the NHANES data to assess potential relationships between outcomes such as obesity and urinary concentrations of various chemicals or their metabolites (Bhandari et al. 2013; Carwile and Michels 2011; Eng et al. 2013; Hatch et al. 2008; LaKind et al. 2012; Melzer et al. 2010, 2012; Trasande et al. 2012; Twum and Wei 2011). In these analyses, urinary concentrations are implicitly assumed to be direct surrogates for relevant chemical exposure levels.

Identification of relevant exposure metrics and interpretation of urinary analyte concentrations in that context requires consideration of mode of action of the chemical of interest as well as the pharmacokinetic and physiological factors that influence the relationship between exposure and urinary concentrations. For chemical risk assessment, systemic exposures as assessed in nanograms chemical per kilogram body weight per day are often used as the metric of interest. However, for chemicals producing toxicity directly to a tissue at the route of entry, other exposure metrics such as non-body weight–adjusted exposure in nanograms per day, may also be of interest. Interpretation of exposures for either of these metrics using urinary concentrations of analytes requires assumptions or data about urinary flow rates. In studies relying on urinary biomarker concentrations for exposure characterization, including most analyses of biomarker concentrations and health outcomes based on the NHANES data sets, biomarker concentrations are usually regarded as direct surrogates for exposure level. Equation 1 shows that daily chemical intake per kilogram body weight (*BW*) (intake rates, IR milligram per kilogram BW) is estimated based on measured concentration (*C*) using generic assumptions about average urinary flow rates (*V*_24_) and a direct proportionality factor that represents the fraction of ingested compound excreted in urine [*FUE*; reviewed by Angerer et al. (2011)]:

*IR =* (*C* × *V*_24_)/(*FUE* × *BW*). [1]

Although sources of random variation in urinary concentrations from spot urine samples, apart from differences in exposure level, are often acknowledged, potential sources of systematic bias are generally not discussed or recognized. One source of variation that has long been recognized is the substantial variation in urinary flow rate as a reflection of hydration status (Barr et al. 2005; Garde et al. 2004). Such variation in flow leads to variation in analyte concentration in urine due to varying dilution of urine, and the flow rate can vary substantially within individuals, within and across days, as well as across individuals.

As a result, a number of approaches to “correcting” urinary concentrations for hydration status have been used. The most common of these is creatinine correction, in which the measured concentration of the analyte is divided by the concentration of creatinine in the urine. This is done with the understanding that creatinine excretion in urine occurs at a rate that is less variable than the rate of urinary flow in volume excreted per time. However, it has been recognized that creatinine excretion rates differ systematically across the population, with children excreting less creatinine per kilogram body weight than adults (Barr et al. 2005; Remer et al. 2002), and with systematic differences between lean and obese individuals and across different racial and ethnic groups, partly attributable to variations in dietary pattern (Mage et al. 2008). Because of these systematic differences, assessment of potential associations between health outcomes and creatinine-corrected biomarker concentrations are subject to potential confounding or systematic bias if the health outcome is also independently associated with these characteristics.

Other sources of variation also exist, and are generally treated as sources of random variation, including variation within and between individuals and within day due to rapid elimination kinetics and timing of sampling relative to exposure (Aylward et al. 2012). This source of variation is not addressed in the present analysis but has been analyzed elsewhere (Aylward et al. 2012; Pleil and Sobus 2013; Preau et al. 2010; Tan et al. 2007).

The most recent surveys released by NHANES (2009–2010, 2011–2012) collected data from each participant on urinary flow, which directly addresses the issue of hydration status without requiring use of an indirect surrogate such as creatinine concentration. Specifically, the time and volume of the complete void(s) collected at the mobile examination center (MEC) were measured, and participants were asked to record the time of their last urinary void before the void collected at the MEC. The measured urinary composite void volume (*V*, milliliters), the recorded time since last void (*t*, hours), the participant body weight (*BW*, kilograms) and the urinary concentration of an analyte (*C*, nanograms per milliliter) can be used to calculate directly the urinary excretion rate (*ER*) of the analyte over the time period covered by the collected urinary void in nanograms per hour:

*ER*(*ng/hr*) *=* (*C* × *V*)/*t*, [2]

or in nanograms per kilogram BW per hour:

*ER*(*ng/kg-hr*) *=* (*C* × *V*)/(*t* × *BW*). [3]

The calculated analyte excretion rate directly accounts for hydration status variations by incorporating the actual urinary flow rate (volume per time either absolute or per kilogram body weight), rather than an assumed “average” flow rate or surrogate factor such as creatinine concentration. This calculation represents the “true” excretion rate of the analyte in nanograms per hour or nanograms per kilogram BW per hour over the time period covered by the urinary spot sample. The factor representing the proportionality constant between the actual excretion rate and the measured concentration of any analyte in urine for a given sample is the urinary flow rate (UFR, milliters per hour) or the body weight–adjusted urinary flow rate (UFRBW, milliters per kilogram per hour).

In the ideal case, even with random variation in urinary flow rates, urinary concentrations would be proportionally and consistently related to analyte excretion rates without systematic differences by age, sex, race/ethnicity, or characteristics such as body mass index (BMI). That is, ideally, the UFR or UFRBW term in Equations 2 and 3, although variable within and between individuals, would be relatively consistent on average across different population groups and characteristics without systematic differences. In that case, urinary concentrations could be used as a direct and unbiased surrogate for excretion rate, and therefore for intake rates, without confounding due to systematic differences in urinary flow. However, if systematic differences in urinary flow rate among groups exist, differences in urinary concentrations would be affected not only by differences in analyte excretion rate due to differences in exposure levels, but also by systematic differences in urinary flow rates. This could confound interpretation of observed associations between outcomes and urinary concentrations in the NHANES data set and in other studies relying on urinary biomarker concentration as an exposure metric. This is particularly true if the urinary flow rates systematically vary along with the health outcome of interest (e.g., obesity).

In this paper we provide an assessment of patterns in UFR and UFRBW (milliters per hour and milliters per kilogram per hour) by age, sex, race/ethnicity, and BMI based on the NHANES 2009–2010 and 2011–2012 data sets. We also provide brief case studies demonstrating the use of unadjusted concentration, creatinine-adjusted concentration, osmolality, and calculated excretion rates (Equations 2 and 3) to examine patterns in exposure to bisphenol A (BPA) and 2,5-dichlorophenol (25DCP) across population groups, with particular focus on patterns with BMI. The results can inform the design and analysis of studies examining the relationship between exposure to chemicals (as measured by urinary biomonitoring data) and potential health outcomes.

## Methods

This analysis relies on data collected during the 2009–2010 and 2011–2012 NHANES survey cycles. Data sets on urinary flow, demographic variables, body measures, urinary creatinine concentration, urinary osmolality, and environmental phenols were downloaded from the NHANES website [Centers for Disease Control and Prevention (CDC) 2014]. Information on participant consent and descriptions of the laboratory methods used for the determination of creatinine, osmolality, and chemical measures are available at the NHANES website (CDC 2014). All statistical analyses were conducted incorporating the NHANES survey design characteristics and appropriate population weights in Stata IC 12.1 (StataCorp, College Station, TX).

*Urinary flow rates*. Urinary flow rate was measured in participants ages 6 and above attending an examination at one of the MECs as part of NHANES during years 2009–2012. The entire urinary void volume was measured and the time of sample collection was recorded at the MEC. In addition, participants were asked to record the time of their last urinary void before the void collected at the MEC. This information was requested in the forms filled out by the participant before the MEC appointment and was also prompted via phone call the evening before the appointment (for documentation, see http://www.cdc.gov/nchs/nhanes/nhanes_questionnaires.htm). Finally, when the initial void collected was of insufficient volume for the clinical and laboratory analyses, up to two additional voids were collected during the MEC visit, with volumes and timing measured, and all collected samples were composited. All laboratory analyses were conducted on the composited sample.

The composite UFR (milliters per hour) was calculated as quotient of the total volume collected and the total time covered by all collected voids, and the UFRBW was calculated by dividing the sample UFR (milliters per hour) by the measured body weight (kilograms) for each participant. The geometric means (GM) [95% confidence interval (CI)] of the UFR and UFRBW were calculated for each age group (6–11, 12–19, and ≥ 20 years) and among adults in age groups 20–39, 40–59, and ≥ 60 years; by sex; category of BMI (< 20, 20 to < 25, 25 to < 30, and ≥ 30); and race as coded by NHANES (Mexican American, non-Hispanic white, non-Hispanic black, and other). Statistical significance of differences in the GMs compared with the reference category for each parameter was assessed using the adjusted Wald test, which is a modified version of the *F*-test that, as implanted in Stata, takes into account the survey design characteristics of the data set.

Use of BMI as a marker for obesity depends on the measured body weight, as does the UFRBW. Therefore, the relationship between UFR or UFRBW and obesity was assessed using a second, independent, measure of obesity: waist circumference. Osmolality was also measured in NHANES using freezing point depression osmometry (for details on laboratory methods, see http://www.cdc.gov/nchs/nhanes). Osmolality is a measure of urine concentration, and associations between urine osmolality and BMI were also examined.

*Analyte case studies*. The effect of consideration of urinary flow rate on exposure assessment was illustrated through examination of alternative methods of using the available biomarker concentration data to characterize exposure. We selected chemicals for which previous outcome association analyses relying on patterns in urinary concentration had been published and for which urinary concentration data were already released for the NHANES 2009–2010 and 2011–2012 data sets. BPA has been the subject of a number of analyses using previous NHANES data sets (without available flow information) and was deemed to be of significant interest (Bhandari et al. 2013; Carwile and Michels 2011; Casey and Neidell 2013; Eng et al. 2013; LaKind et al. 2012; Melzer et al. 2010, 2012; Trasande et al. 2012). Twum and Wei (2011) reported on associations between urinary concentrations of 25DCP and obesity in children and adolescents, and we selected this as a second example. Urinary concentration data were assessed here for BPA and 25DCP for selected age groups. We examined the impact of quantifying exposures in terms of unadjusted concentrations, creatinine- or osmolality-adjusted concentrations, and calculated analyte mass excretion rate (nanograms per hour or nanograms per kilogram per hour) on patterns across BMI category.

We calculated GM analyte concentrations by BMI category within age groups for the example analyses based on unadjusted measured concentration, creatinine-corrected and osmolality-adjusted concentrations, and based on excretion rate (equations 2 and 3). Creatinine-corrected concentrations (*C_cr-adj_*) were calculated as

*C_cr-adj_ = C_vol_*/*C_cr_*, [4]

where *C_vol_* is the measured volume-based concentration of the analyte in nanograms per milliter, and *C_cr_* is the creatinine concentration in grams per milliter. Osmolality-adjusted concentrations (*C_osm-adj_*) were calculated as (Frederiksen et al. 2013)

*C_osm-adj_ =* (*C_vol_* × *Osm_med_*)/*Osm_meas_*, [5]

where *Osm_med_* is the median population osmolality, and *Osm_meas_* is the measured osmolality in a specific sample. Urinary specific gravity is also sometimes used to correct for hydration status, but this was not measured in the NHANES 2009–2012 data sets and so is not assessed here.

## Results

**Figure 1:**
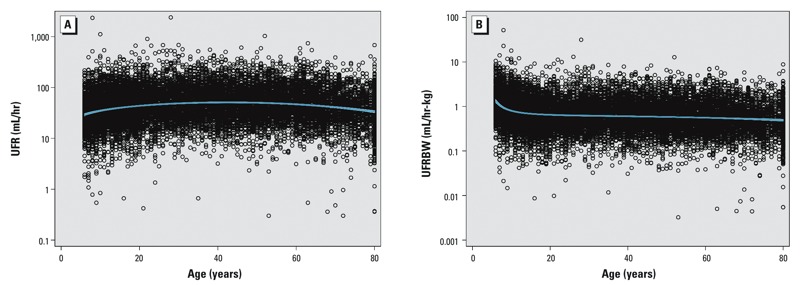
UFR (mL/hr) (*A*) and UFRBW (mL/hr-kg) (*B*) as a function of age in the NHANES 2009–2012 data sets. Blue line is the fractional polynomial fit with 95% CI to the logarithm of urinary flow rate vs. age.

UFR data were available for 14,631 participants in the 2009–2012 NHANES cycles. Summary statistics on the distributions of collected void volumes and time covered by the collected urine samples by age group are presented in Table 1. The GMs for UFR and UFRBW across the entire 2009–2012 NHANES data set were 47.76 mL/hr (95% CI: 45.64, 49.98 mL/hr) and 0.65 mL/hr-kg (95% CI: 0.63, 0.67 mL/hr-kg), respectively. Patterns of UFR and UFRBW with age were assessed visually and notable differences among age groups were observed (Figure 1). UFR as a function of age rises through childhood and into adulthood and then declines in older adults. UFRBW exhibits a steep decline in children < 12 years of age, with a more gradual decline with age after 12 years. As a result, subsequent assessments relied upon age stratification.

**Table 1:** Summary statistics (mean and key percentiles) for collected void volumes and time covered by collected void volumes.

Age group (years)	*n*	Mean	p5	p25	p50	p75	p95
First collected void volume (mL)
6–11	2,014	93	13	36	69	128	259
12–19	2,251	122	17	44	94	178	324
20–39	3,627	128	19	50	103	182	324
40–59	3,499	120	21	54	96	163	309
≥ 60	3,356	101	14	41	78	138	264
Composite collected volume (mL)
6–11	2,014	110	30	55	89	146	267
12–19	2,251	150	41	74	124	205	337
20–39	3,627	155	48	82	129	210	341
40–59	3,499	136	40	69	111	178	325
≥ 60	3,356	122	34	63	99	159	287
Time since previous void for first collected void (min)
6–11	1,993	178	48	90	141	213	444
12–19	2,231	192	47	87	149	222	582
20–39	3,604	159	43	81	128	192	375
40–59	3,491	138	42	75	117	170	296
≥ 60	3,334	135	42	76	116	173	277
Total time covered by composited voids (min)
6–11	2,014	194	58	110	165	225	459
12–19	2,251	214	60	118	178	245	582
20–39	3,627	182	55	104	160	218	386
40–59	3,499	153	46	88	131	191	317
≥ 60	3,356	159	52	94	145	206	301
p, percentile.							

Figure 1 demonstrates that a few samples displayed very low or very high flow rates. Conventionally, such extreme values are omitted from assessment of biomonitoring data sets (when UFR data are available) (Santonen et al. 2014). It is possible that these values reflect errors in reported time since last void or void collection volumes. However, because the collections are spot samples rather than 24-hr collections, substantial variation in UFR is possible. The results reported here were not affected by trimming the data set to remove the most extreme values.

UFR was significantly greater in males than in females for all age categories except children 6–11 years of age (Table 2). In contrast, UFRBW did not differ between males and females at any age (Table 3).

**Table 2:** GM UFR (mL/hr) from NHANES 2009–2012 by age group, sex, race/ethnic group, and BMI categories.

Mean	6–11 years		12–19 years		20–39 years		40–59 years		≥ 60 years	
	GM (95% CI)	*p-*Value^*a*^	GM (95% CI)	*p-*Value^*a*^	GM (95% CI)	*p-*Value^*a*^	GM (95% CI)	*p-*Value^*a*^	GM (95% CI)	*p-*Value^*a*^
All	33.3 (31.4, 35.3)	< 0.001^*b*^	42.5 (40.7, 44.4)	< 0.001^*b*^	51.1 (48.5, 53.8)	Reference	51.9 (50.2, 53.6)	0.6^*b*^	43.2 (41.5, 44.9)	< 0.001^*b*^
Sex
Male (reference)	33.6 (31.6, 35.9)	Reference	44.5 (42.0, 47.2)	Reference	54.5 (51.9, 57.3)	Reference	55.1 (52.5, 57.9)	Reference	47.4 (44.7, 50.2)	Reference
Female	33.0 (30.3, 35.9)	0.7	40.5 (38.1, 43.0)	0.02	47.9 (44.7, 51.4)	< 0.001	49.0 (47.2, 50.8)	< 0.001	40.0 (37.9, 42.3)	< 0.001
Race/ethnic group
Mexican American	31.5 (28.7, 34.7)	0.1	41.9 (38.7, 45.5)	0.3	44.8 (41.5, 48.3)	< 0.001	43.8 (40.4, 47.4)	< 0.001	37.5 (33.0, 42.6)	0.02
Non-Hispanic White	35.0 (32.0, 38.3)	Reference	44.3 (41.3, 47.6)	Reference	55.7 (52.3, 59.2)	Reference	54.9 (52.7, 57.2)	Reference	44.3 (42.3, 46.5)	Reference
Non-Hispanic Black	31.1 (28.1, 34.4)	0.09	36.9 (33.8, 40.2)	< 0.001	38.8 (36.9, 40.9)	< 0.001	42.5 (40.3, 44.9)	< 0.001	37.9 (35.9, 40.0)	< 0.001
Other	33.9 (28.9, 39.8)	0.7	43.9 (39.7, 48.7)	0.9	52.9 (48.7, 57.5)	0.2	52.3 (45.9, 59.5)	0.5	41.6 (36.4, 47.6)	0.3
BMI
< 20 (reference)	32.2 (30.0, 34.6)	Reference	40.6 (37.0, 44.5)	Reference	46.0 (39.5, 53.6)	Reference	54.4 (44.7, 66.3)	Reference	39.0 (32.3, 47.2)	Reference
20 to < 25	37.3 (32.9, 42.3)	0.04	42.4 (39.6, 45.5)	0.4	52.6 (48.4, 57.1)	0.09	56.5 (52.1, 61.1)	0.7	41.4 (37.4, 45.8)	0.6
25 to < 30	38.4 (33.5, 44.1)	0.03	43.5 (39.7, 47.6)	0.3	52.1 (47.6, 57.1)	0.2	52.1 (48.8, 55.6)	0.7	45.3 (41.7, 49.3)	0.2
≥ 30	25.7 (19.8, 33.3)	0.1	45.2 (40.7, 50.1)	0.1	49.7 (46.9, 52.7)	0.3	48.9 (47.1, 50.8)	0.3	42.6 (40.5, 44.9)	0.4
^***a***^*p*-Values present comparison to reference group within each age stratum for each categorization (sex, race/ethnic group, or BMI category), except as otherwise indicated. ^***b***^Compared with ages 20–39 years.										

**Table 3:** GM UFRBW (mL/hr-kg) from NHANES 2009–2012 by age group, sex, race/ethnic group, and BMI categories.

Mean	6–11 years		12–19 years		20–39 years		40–59 years		≥ 60 years	
	GM (95% CI)	*p-*Value^*a*^	GM (95% CI)	*p-*Value^*a*^	GM (95% CI)	*p-*Value^*a*^	GM (95% CI)	*p-*Value^*a*^	GM (95% CI)	*p-*Value^*a*^
All	1.01 (0.95, 1.07)	< 0.001^*b*^	0.67 (0.63, 0.70)	0.5^*b*^	0.65 (0.62, 0.69)	Reference	0.64 (0.62, 0.66)	0.4^*b*^	0.56 (0.53, 0.58)	< 0.001^*b*^
Sex
Male (reference)	1.04 (0.97, 1.11)	Reference	0.66 (0.61, 0.71)	Reference	0.64 (0.61, 0.67)	Reference	0.62 (0.59, 0.65)	Reference	0.56 (0.53, 0.59)	Reference
Female	0.98 (0.89, 1.07)	0.3	0.67 (0.63, 0.72)	0.7	0.66 (0.61, 0.72)	0.3	0.66 (0.63, 0.68)	0.04	0.55 (0.52, 0.59)	0.7
Race/ethnic group
Mexican American	0.94 (0.85, 1.03)	0.02	0.67 (0.62, 0.72)	0.5	0.57 (0.53, 0.62)	< 0.001	0.55 (0.51, 0.60)	< 0.001	0.49 (0.43, 0.56)	0.05
Non-Hispanic White	1.08 (0.98, 1.19)	Reference	0.70 (0.64, 0.75)	Reference	0.71 (0.66, 0.75)	Reference	0.67 (0.64, 0.70)	Reference	0.57 (0.54, 0.60)	Reference
Non-Hispanic Black	0.87 (0.78, 0.97)	0.005	0.55 (0.51, 0.59)	< 0.001	0.46 (0.43, 0.49)	< 0.001	0.49 (0.46, 0.51)	< 0.001	0.47 (0.44, 0.49)	< 0.001
Other	1.09 (0.95, 1.25)	0.9	0.72 (0.64, 0.82)	0.6	0.75 (0.69, 0.83)	0.1	0.75 (0.65, 0.87)	0.1	0.61 (0.52, 0.71)	0.3
BMI
< 20 (reference)	1.12 (1.05, 1.21)	Reference	0.84 (0.77, 0.91)	Reference	0.87 (0.75, 1.00)	Reference	1.06 (0.86, 1.31)	Reference	0.80 (0.68, 0.95)	Reference
20 to < 25	0.85 (0.75, 0.96)	< 0.001	0.69 (0.64, 0.74)	< 0.001	0.81 (0.75, 0.87)	0.4	0.87 (0.80, 0.95)	0.06	0.66 (0.60, 0.73)	0.04
25 to < 30	0.65 (0.57, 0.75)	< 0.001	0.57 (0.52, 0.63)	< 0.001	0.66 (0.61, 0.72)	0.004	0.66 (0.62, 0.70)	< 0.001	0.59 (0.55, 0.64)	0.004
≥ 30	0.43 (0.35, 0.52)	< 0.001	0.46 (0.41, 0.51)	< 0.001	0.49 (0.46, 0.52)	< 0.001	0.48 (0.46, 0.50)	< 0.001	0.45 (0.43, 0.48)	< 0.001
^***a***^*p*-Values present comparison with reference group within each age stratum for each categorization (sex, race/ethnic group, or BMI category), except as otherwise indicated. ^***b***^Compared with ages 20–39 years.										

Complex patterns by race and ethnicity were observed for both UFR and UFRBW (Tables 2 and 3). Compared with non-Hispanic white (NHW) participants, Mexican-American (MA) and non-Hispanic black (NHB) adult participants had significantly lower UFR and UFRBW. UFR and UFRBW in NHB children and adolescents were also significantly lower than in NHW children and adolescents. UFRBW was lower in MA children than NHW children. Participants identified as “other” did not differ significantly from NHW participants in any age group for either metric.

With respect to BMI, UFR in children increased in the middle two BMI categories compared with the lowest BMI category, and was markedly lower in the BMI > 30 category compared with BMI < 20 (Table 2). For adolescents and adults, UFR was essentially independent of BMI category (Table 2). This implies that urinary flow does not increase proportionally with body weight after childhood. In contrast, UFRBW varied inversely with BMI category in all age groups (Table 3). The magnitude of decrease from the lowest (BMI < 20) to highest category was nearly 3-fold in the youngest age group (from 1.12 to 0.43 mL/hr-kg, *p* < 0.001), and nearly 2-fold in adolescents (0.84 to 0.46 mL/hr-kg, *p* < 0.001) and adults (e.g., 1.06 to 0.48 mL/hr-kg, *p* < 0.001, in adults ages 40–59 years). The magnitude of this variation across BMI categories is larger than the magnitude of differences observed among racial and ethnic groups or across age groups. This inverse relationship is a natural consequence of the observation that UFR is relatively independent of BMI, at least in adolescents and adults: In a given stratum, as body weight and therefore BMI increase, the rate of urinary flow per kilogram body weight declines.

**Figure 2:**
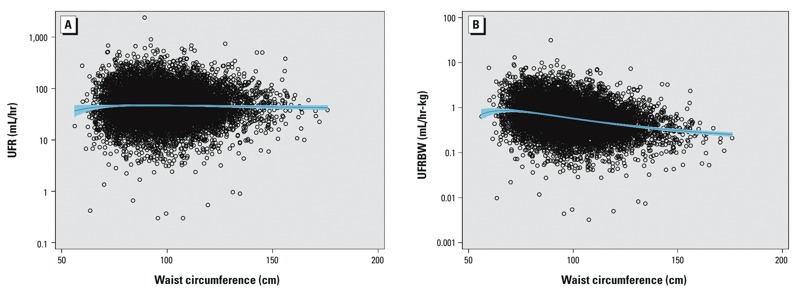
UFR (mL/hr) (*A*), and UFRBW (mL/hr-kg) (*B*) as a function of waist circumference in adults in the NHANES 2009–2012 data sets. The blue line is the fractional polynomial fit with 95% CI to the logarithm of urinary flow rate vs. waist circumference.

Results from the analysis of urinary flow rate as a function of waist circumference (WC) were consistent with the patterns observed with BMI. UFR was essentially independent of WC, whereas UFRBW declines as WC increases (Figure 2A and 2B, respectively).

**Figure 3:**
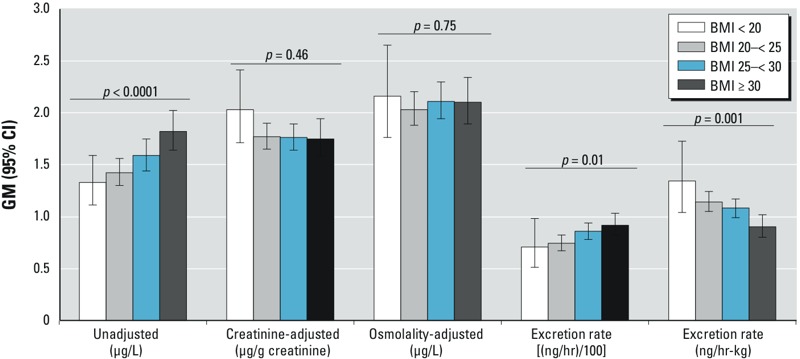
GM (95% CI) urinary BPA concentration, creatinine-adjusted concentration, osmolality-adjusted concentration, and mass excretion rates [(ng/hr)/100 or ng/hr-kg] by BMI category based on urinary spot samples from adults in the NHANES 2009–2012 data sets. Mass excretion rates were plotted divided by 100 to allow comparison of pattern on the same numerical scale with the other metrics. *p*-Values are assessment for trend across BMI categories.

*Analyte case studies*. BPA and BMI in adults. GM urinary concentrations of BPA in adults from the NHANES 2009–2012 survey data (*n* = 3,395) increased monotonically and significantly with BMI category (Figure 3). The association disappears when two methods of correction for hydration status, creatinine and osmolality adjustment, are applied to the measured urinary BPA concentrations. When assessed on the basis of mass excretion rate (nanograms BPA per hour), the pattern of excretion rate by BMI category looks similar to the pattern by concentration. However, on the basis of body weight–adjusted excretion rate, a statistically significant association is again observed, but reversed in direction, with the lowest mass excretion rate for BPA in the highest BMI category.

**Figure 4:**
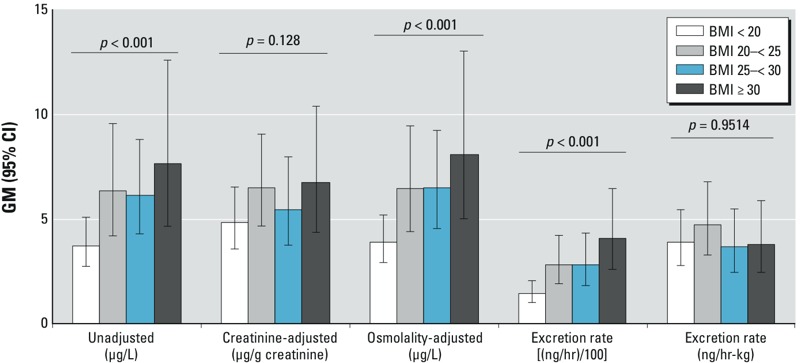
GM (95% CI) urinary 25DCP concentration, creatinine-adjusted concentration, osmolality-adjusted concentration, and mass excretion rates [(ng/hr)/100 or ng/hr-kg] by BMI category in children and adolescents in the NHANES 2009–2012 data sets. Mass excretion rates were plotted divided by 100 to allow comparison of pattern on the same numerical scale with the other metrics. *p*-Values are assessment for trend across BMI categories.

25DCP and BMI in children and adolescents. GM urinary concentrations of 25DCP in children and adolescents also trended upward with BMI category, doubling between the lowest and highest BMI categories (Figure 4). After applying hydration status adjustment using creatinine, the trend became nonsignificant. The trend remained when osmolality adjustment was applied. GM mass excretion rates (nanograms per hour) showed a similar pattern to that for unadjusted concentration, whereas there was no trend in body weight–adjusted excretion rates across BMI categories.

## Discussion

The collection of UFR data in the NHANES examinations beginning in 2009 allows an expanded understanding of factors to be considered in the valid interpretation of urinary biomarker data as a metric of exposure. The conceptual model for interpreting urinary biomonitoring data for rapidly metabolized compounds is based on the recognition that many of these compounds are eliminated (as parent compound or metabolite) primarily in urine, with urinary excretion of analyte (parent or metabolite) directly proportional to the rate of intake of parent compound, on average. Urinary concentrations are usually treated, implicitly or explicitly, as direct surrogates for intake rates. Variations in hydration status have been recognized as an important source of variability in measured urinary concentrations, but this has conventionally been assumed to be a source of random error. The collection of UFR information in the post-2008 NHANES cycles provides a powerful tool for assessing this assumption. The analyses presented here show that there are strong and systematic variations in UFR as a function of age, sex, and race or ethnicity, and strong and systematic variations in UFBBW as a function of age, race or ethnicity, and BMI.

Because measured urinary concentrations are functions of both analyte excretion rate, which is related to chemical exposure levels, and UFR, which is not, systematic variations in UFR associated with health outcomes of interest can directly confound the assessment of associations between urinary analyte concentrations and that health outcome. In the initial analyses presented here, the relative independence of UFR and the resulting systematic inverse variation of UFRBW with BMI is directly relevant to the evaluation of potential associations between urinary analyte concentrations, and not only markers for obesity but also diseases for which obesity may be a risk factor.

Conventional methods for adjustment for hydration status, such as creatinine correction, did not fully address the systematic variations in UFR observed here. Creatinine excretion itself has been recognized to vary systematically in the population, with well-recognized differences among excretion rates in young children, young adults, and older adults (on a per kilogram BW basis as well as on an absolute basis); differences between sexes; variation with BMI, and variations with dietary pattern and renal health status (Barr et al. 2005; Mage et al. 2008; Remer et al. 2002). As a result, Barr et al. (2005) have recommended that urinary creatinine concentration be entered as an independent variable in regression analyses rather than applied as a hydration status “correction” factor to measured urinary analyte concentrations.

However, the systematic variations in UFRBW with BMI observed in the NHANES data set are not necessarily related to hydration status per se. That is, it is not necessarily true that overweight and obese individuals are “dehydrated” relative to lean individuals. Total body water and water replacement requirements are most directly related to fat-free mass. Increases in fat mass have much smaller impacts on total body water and fluid balance than increases in fat-free mass (Chumlea et al. 1999, 2005; Wang et al. 1999), and urinary output rates (milliliters per hour) do not increase linearly with body weight. Thus, methods such as adjustment for specific gravity or creatinine concentration, which implicitly address physiologically “concentrated” or “dilute” urine, may not address the reduced UFR compared with lean individuals (per kilogram body weight) that would be “normal” in obese subjects. This is supported by the examination of urinary osmolality data from NHANES. Urinary osmolality—a measure of particles per mass of urine, and thus of general urinary concentration—increases monotonically with BMI in all age categories (*p* < 0.001, data not shown). This suggests that physiologically, urine is more concentrated on average in persons with higher BMI, and that this is physiologically normal and does not represent a deficient hydration status.

The systematic variation of UFR with BMI could lead to “reverse causation,” in which the health outcome (obesity, or diseases that can be directly associated with obesity) influences the analyte concentration through reduced UFR, rather than analyte concentration causing the health outcome. The further systematic variation of flow rate among categories of race and ethnicity also should be considered in design and analysis of biomonitoring-based studies. Other factors not directly considered here (e.g., fasting time, time of day, time since last void) may also have impacts on UFR that should be assessed for systematic sources of variation in interpretation of biomarker concentrations. However, assessment of exposure on the basis of analyte excretion rate in nanograms per kilogram body weight per hour rather than urinary analyte concentration directly accounts for such variations. UFRs have also recently been shown to partially or fully account for apparent associations between low urinary cadmium concentrations and urinary protein levels (Akerstrom et al. 2013). Further consideration of urinary excretion of target analytes, urinary flow, and potential interrelationships with outcomes of interest should be applied in studies using urinary biomarkers of exposure.

Consideration of UFR and calculated analyte excretion rate does not address other sources of variation in biomarker concentrations or excretion rate. For example, the impact of rapid pharmacokinetics of these compounds, and therefore the impact of the time between exposure and sampling on both urinary concentration and analyte excretion rate, have been recognized. As a result, assessment of within- and between-person variability in biomarker concentrations and evaluation of the potential magnitude of such variation has become an important component of such studies (Aylward et al. 2012, 2014; Preau et al. 2010; Ye et al. 2011).

Finally, reliance on analyte mass excretion rate calculated as the product of urinary flow rate and analyte concentration for exposure classification does not reduce the importance of having a conceptual model of the exposure pathways, routes, and timing, and hypotheses regarding the likely pattern of analyte excretion rates. For example, in studies in which BMI is an outcome of interest, the route of exposure may result in systematic variations in urinary analyte excretion as a function of BMI. For chemicals for which the predominant exposure pathway is dietary, food intake rates may scale reasonably directly with bodyweight, and therefore, the null hypothesis might suggest that equal food intakes (per kilogram body weight) across BMI categories would result in increasing intake of contaminant (nanograms per day) as BMI increases, and therefore increasing mass excretion rates in nanograms per day with BMI. However, on the basis of nanograms per kilogram per day, intakes and excretion rates might be expected to be similar across BMI categories. In contrast, if the primary exposure pathway is inhalation, for the same concentration in air, persons with higher BMI may inhale less air per kilogram body weight and therefore less compound per kilogram body weight (Brochu et al. 2014), and so might be expected to have lower analyte excretion rates (nanograms per kilogram per day) compared with lean individuals.

The differences in absolute pattern of concentrations and excretion rates across BMI categories for the two case-study chemicals presented here may be attributable to such differences in exposure patterns. However, in both cases, the pattern across BMI categories for concentration versus body weight–adjusted excretion rate changed in the same direction, reducing or reversing the positive association between concentration and BMI category.

This analysis presents an initial evaluation of patterns in UFR across age, sex, race/ethnicity, and BMI using simple comparison of geometric mean flow rates in the NHANES 2009–2012 data sets. These data sets provide a wealth of data that can and should be examined with further evaluations to inform the understanding of the effect of physiological and sampling characteristics on urine spot samples. Such factors might include, but not be limited to, time of day of sampling, reported fasting time, glomerular filtration rate, and detailed patterns with age for children in the 6- to 11-year-old age range. In particular, an examination of glomerular filtration rate and urinary flow characteristics can potentially inform how patterns in UFR, and resulting patterns in urinary concentration of analytes may be related to blood concentrations of the same chemical, at least for those chemicals that are excreted via filtration. The data sets can also be used to inform distributions and covariation assumptions included in detailed physiologically based pharmacokinetic modeling that addresses urinary excretion of chemicals. In addition, the patterns in UFR presented here may also influence the interpretation of urinary concentrations observed in populations monitored for occupational exposures in relationship to Biological Exposure Indices (American Conference of Governmental and Industrial Hygienists 2014) or other occupational biomonitoring guidelines.

The strong and significant systematic variations of UFR observed across categories of age, race/ethnicity, and BMI suggest that variation in urinary analyte concentrations due to “hydration status” should not be assumed to be random with respect to health outcomes or populations of interest. Further, conventional methods for addressing “hydration status” are insufficient for accounting for the observed systematic variation in flow rates. The case studies presented here are not meant to be exhaustive assessments of the associations reported between various analytes and BMI or other characteristics, but rather to illustrate the potential impact of consideration of flow rate. The results presented here suggest that future studies should examine this issue carefully when assessing associations between exposure and response using biomonitoring data, in order to reduce the potential for associations that represent “reverse causation.”

The analyses here suggest that future evaluations of potential associations between health outcomes and chemical exposures as reflected in urinary biomonitoring data should be assessed in the NHANES survey post-2008 not only on the basis of biomarker concentrations, but also on the basis of mass excretion rate (nanograms per hour and nanograms per kilogram per hour). Such evaluations should be structured using clear hypotheses regarding the relationship between exposure pathway, exposure metric, and health outcome of interest. Previous analyses of the cross-sectional NHANES data sets for associations between urinary concentrations of environmental chemicals and, for example, measures of obesity or health outcomes such as cardiovascular disease for which BMI is a risk factor (e.g., Bhandari et al. 2013; Carwile and Michels 2011; Eng et al. 2013; Hatch et al. 2008; LaKind et al. 2012; Melzer et al. 2010, 2012; Trasande et al. 2012; Twum and Wei 2011), should be reassessed in the post-2008 NHANES data sets using the available flow rate data to calculate analyte mass excretion rates and be considered in this framework. Finally, in studies outside the NHANES framework that rely on spot urine samples, consideration should be given to collection of additional data on time of last urinary void and total void volume. These data will allow calculation of analyte excretion rate and permit evaluation of potential associations with health outcomes on the basis of a more direct exposure metric as well as on the basis of urinary concentration.
